# Diphtheria and Its Treatment: Homœopathic Views

**Published:** 1883-04

**Authors:** Ad. Lippe

**Affiliations:** Philadelphia


### DIPHTHERIA AND ITS TREATMENT: HOMŒOPATHIC VIEWS.

AD. LIPPE, M. D., PHILADELPHIA.

We find in a Philadelphia paper the following publication of the proceedings of the Philadelphia County Homœopathic Society:

DIPHTHERIA AND ITS TREATMENT—HOMŒOPATHIC VIEWS.—At the monthly meeting of the Homœopathic Medical Society of the County of Philadelphia, last night, Dr. Martin presiding, there was a special discussion of the subject, "The Treatment of Diphtheria." Dr. C. Mohr, who opened the discussion, said that he had, in his experience, had seventeen cases of the disease under treatment at one time, and of that number two had died. He had used various applications, but had come to regard the Chlorinated Lime and Alcohol with most confidence. The latter, he thought, was the most efficacious agency that could be employed in destroying the bacteria, or micrococci. As to the cause of the disease, he believed it to be a blood poisoning, and that the bacteria are the result and not the source of the disease; but that, even if the germ theory were correct, no general germicide or parasiticide need be looked for, as each case, even then, must be individualized and treated with remedies specially suited to itself. In reply to questions, Dr. Mohr said that he used the Alcohol as a gargle, with a ten per cent. solution in water, and when a child was too young to use it in that way he applied it with a camel's hair brush.

Dr. Morgan remarked that he had used Aconite and Belladonna with uniform success in diphtheria while practicing in Alton, Illinois, but found less happy results from the same remedies upon removing to Lebanon. He also had found that Permanganate of Potash was used with excellent effect in cases where the false membrane was yellow, while it utterly failed when there was a white membrane, and that Phylolaca was efficient in cases with white membrane, but not so much so in cases where the membrane was yellow.

Dr. Guernsey, who was detained by illness, sent a written communication, which was read, and in which he recounted the details of a severe case, in which Bromine in a dry form, and afterward Lactomenim [Lac. caninum ?] had brought about a rapid and complete cure.

Dr. Dudley, referring to some observations previously made on the use of mercurial remedies, said he had employed the red Iodide of Mercury almost uniformly in cases of diphtheritic sore throat and true diphtheria, and had almost always had good results. He had never seen a case of laryngal diphtheria follow its early administration, and, what was more, could count on the fingers of one hand all the bad cases of diphtheria he had ever had. He attributed to the Iodide of Mercury (red) the virtue of nipping the disease in the bud.

Dr. Allen, of Frankford, said he had found the Kali-b. quite a specific this winter in diphtheria, though it had failed in former epidemics of the disease.

Dr. Martin had found reasons for relying more upon Belladonna, in conjunction with the Alcohol treatment, than upon any other remedy for this disease, and related the circumstances of an apparently hopeless case in which it had been successfully used by him.

A paper sent by Dr. Neidhard was read by Dr. Wilcox, in which the former after speaking of the disease and its nature, recommended Chlorinated Lime as a specific, the Lime to be used internally, and as an external application, with Gum Arabic to disguise its offensive taste when administered internally. Several physicians present participated in the further discussion of the subject.—*Public Ledger, February 9th, 1883.*

*Comments:* The first question presenting itself is, What was the motive of this publication? Were the good people of the City of Brotherly Love to be informed *officially* what were the true homœopathic views about diphtheria and its treatment? Was this official communication made by order of the County Society with the full knowledge that a general consent by all homœpathists in the city or in the country could not be given to the views expressed? Has not this publication stultified the men who desired to parade themselves as exponents of true Homœopathy?

The gentleman who opened the discussion declares that he had at *one time* seventeen cases of diphtheria under treatment, and only lost two of them. There is probably not one physician in this city who ever had seventeen cases of diphtheria on hand at one time; some men of extensive practice do not see seventeen cases of true diphtheria in a year; some have not even seen seventeen cases in five years. This gentleman believes, as do most medical men of all schools of medicine, that the bacteria are the result, not the cause, of the disease; if that is so, why does he want to kill them with alcohol? Where is the logic? Where does he find it sustained by any teachings of the homœopathic healing art? The *recovery* is quite possible if the bacteria are destroyed by alcohol, but a *cure* is not quite certain; while a permanent, mild, and safe cure is a positive result if the case, even of very malignant diphtheria, is treated in every sense homœopathically. He first says that he had come to regard the chlorinated lime and alcohol with most confidence, and later he falls out of his text and utters a homœopathic sentence—each case must be individualized and treated with remedies specially suited to itself. When and under what circumstances does he find the individualized indications for chlorinated lime and alcohol? Pardon, sir!—the indications given by this homœopath is "most confidence." An elegant phraseology indeed—first generalize on "confidence" and then play the homœopath and recommend individualization! Such is the lack of consistency and logic paraded before the public in "newspapers." The next speaker declares, what everybody knows, that the remedies indicated in one epidemic at a certain time and in a certain place may not be the specifics for the similar form of disease at another time and in another place; he gives two indications, permanganate of potash where the membrane is *yellow*, and Phytolacca where it is *white*. Remember the keynote, and prescribe for *one* symptom—that is science. Next we had the relation of a case in which Bromine was given or used in a dry form, and afterward "Lactomenim." [Lac. caninum?] We hope to see this case published in a jounal.

The next speaker sends speaker No. 1 high and dry; says he could count on the fingers of one hand all the bad cases of diphtheria he ever had. Speaker No. 1 had seventeen at one time! This gentleman's great remedy is the red Iodide of Mercury.

Next comes a gentleman who informs us that he found this season Kali bichr. almost a specific, although in other epidemics it had failed him. Fine talk indeed! What were or rather what are the indications for Kali bichr.? Or is it proper and right for a healer to use this or the other remedy empirically without indications? Does not the blind hen find a little grain sometimes?

The next man stated that he had reasons for relying upon Belladonna in conjunction with the alcohol treatment, and makes good the assertion of his discovery by relating one *desperate* case.

To crown the ridiculousness of the former declarations, a paper was read by another discoverer, who calls himself a homœopath—in spite of all the assertions made by the founder of our healing art, that the true healer NEVER treats the name of a disease; secondly, that every case is a case per se, and thirdly, that no specific diseases can possibly exist. This philosopher *professes* to have found a specific for diphtheria; he is also the inventor of a specific for yellow fever and for hooping cough. The *specific* for diphtheria, according to *that* authority, is chlorinated lime, to be used internally and externally.

The publication of the above report of the views of the Philadelphia County Homœopathic Society as to the treatment of diphtheria, containing, as it does, sentiments in opposition to the well-known tenets of the homœopathic healing art, was not wise at a time when the opponents of our school, "the allopathists," are diligently collecting evidence to show that Homœopathy, *as taught by Hahnemann*, is extinct; it must also be remembered that the allopathic physicians, as well as a large number of the intelligent public, are fully conversant with the laws governing the homœopathic school of medicine, and while the former receive much desired material, showing plainly the departures from the school, the latter must feel mortified to find some men, who still profess to be homœopathists, go before the public flaunting their individual misconceptions of a school to which they profess to belong, knowing, as they do, that they utter sentiments not indorsed by a large number of honest and consistent homœopathicians.

We now take up the previous question (*Diphtheria and its Treatment; Homœopathic Views*). The homœopathic views of the treatment of diphtheria have been so well expressed in a little work by Dr. R. R. Gregg that we might merely refer to it, but as it is apparent that the members of the Philadelphia County Homœopathic Society recently ventilating their views on that subject cannot be aware of the existence of such a work, and as they have also displayed gross ignorance of the fundamental principles governing the homœopathic healing art and the rules guiding us in applying said fundamental principles in therapeutics, we shall attempt to give a synopsis of them.

The first duty of the healer is to take a true picture of the case to be treated, be it diphtheria or any other disease. We first find the locality, next the kind of affection, the concomitant symptoms, and the conditions under which they appear, just as we are taught in the *Organon*, paragraphs 84 to 104. And that we may correctly apply the *only* law of cure, the law of the similars, we search among the proved drugs for the similar remedy. The latter is by far the most difficult task. The single remedy and the single dose should always be administered first, irrespective of low or high potencies; that choice must always be left to the individual judgment of the healer, who may be guided by his own experience as well as by the experience of others. One of the great rules is *never* to repeat *that* single dose till its effects are exhausted; the graver the disorder the more disastrous will be the results if that golden rule is violated.

The inventor of the assertion that chlorinated lime is a specific for diphtheria argues that the chemical components of chlorinated lime separately show by their provings that they each cover almost all the known diphtheria symptoms (an error), and, therefore, if chemically combined will cure *all* cases; that compound to be a specific. Just as well might this illogical inventor keep his diphtheria bottle open and add every remedy which anybody recommends as a specific for diphtheria, mix them all up as *the* specific for diphtheria. The law of the similars can never be misapplied in this manner, nor can homœopathists indorse pathological picture-books.

